# Thermally activated processes: the underlying mechanism of activated state formation†

**DOI:** 10.1039/d4ra06983h

**Published:** 2024-12-11

**Authors:** Maksym M. Lazarenko, Yuriy F. Zabashta, Oleksandr M. Alekseev, Sergei A. Alekseev, Kateryna S. Yablochkova, Liena Yu. Vergun, Dmytro A. Andrusenko, Konstantin V. Cherevko, Victoria B. Shevchenko, Roman V. Dinzhos, Leonid A. Bulavin

**Affiliations:** a Taras Shevchenko National University of Kyiv 64, Volodymyrska Street Kyiv UA 01601 Ukraine lazmaxs@knu.ua; b Petro Mohyla Black Sea National University Mykolayiv Ukraine

## Abstract

In the present manuscript, we highlight the contradictions in the thermally activated processes theory which treats a system's activated state as a state of the phonon subsystem. We offer an alternative model, in which the activated state is treated as an electron subsystem state. The mechanism of the activated state formation is as follows: thermal fluctuations excite electrons of some particles within the activation zone. This excitation is then shared with other particles in the ground state. This creates a locally-equilibrium activated state. We estimate the lifetime of such a state and derive expressions for the activation energy and entropy, necessary to calculate the number of excited particles in the activation zone and the energy of the particle's excitation. We validate the model experimentally, by examining the behavior of nanocrystals of undecylenic acid in pores of silica gels using dielectric spectroscopy and the analysis of the complex dielectric permittivity behavior at different temperatures and with different frequencies of the external field. The estimated number of excited particles in the activation zone of the nanocrystals and the particle excitation energy for the dielectric relaxation process observed in undecylenic acid confirm that the results of the experiment align well with the proposed model.

## Abstract 2

In the present manuscript, we highlight the contradictions in the thermally activated processes theory which treats a system's activated state as a state of the phonon subsystem.

## Graphical abstract

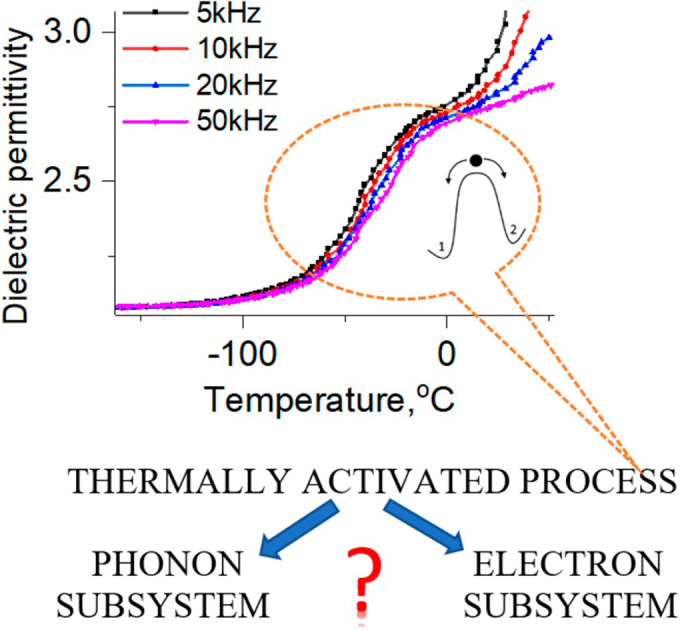

## 1. Introduction

### 1.1. Thermoactivated processes in thermodynamics: the problem with the pre-exponential time

The range of physical parameters, which are traditionally referred to as thermally activated, is very broad: from the chemical reactions^1^ to various diffusion in condensed matter processes.^2^ The array of experimental methods applied to tackle these problems is similarly diverse. They include NMR, PMR, neutron scattering, as well as mechanical and dielectric relaxation methods.^3–11^ Regardless of the method applied, the ultimate validation of theoretical framework used to describe the thermally activated physical properties lies in its concordance with experimental data. Divergence between theory and experiment always necessitates a reevaluation of the underlying assumptions or the introduction of refinements to the model.

Thermodynamics (see ref. 2, among other sources) defines a thermally activated process as the sequence of a system's states whose free energies form a sequence:1
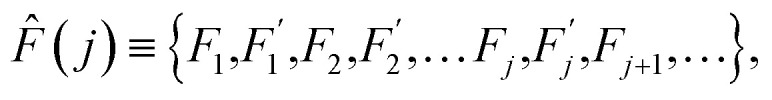


which satisfies the following condition:2
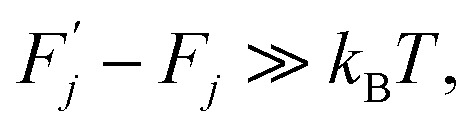
3
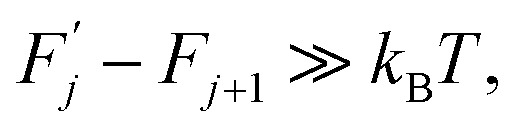
where *k*_B_ is Boltzmann's constant and *T* is temperature.

Within this sequence, a transition between the successive states4
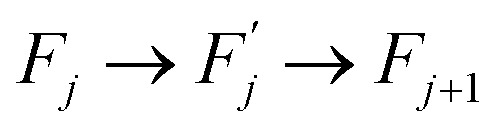


is usually referred to as an elementary thermal fluctuation event.

Expression (4) implies that during the transition the system must overcome an energy barrier:5
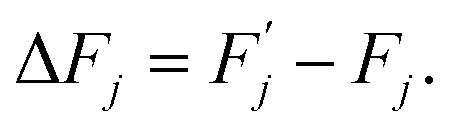


This barrier is overcome by means of thermal fluctuations. Consequently, the random process *F̂*(*j*) is referred to as a thermally activated process, the state with free energy 
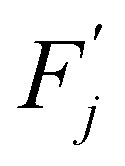
 as activated, while the state with free energy *F*_*j*_ as inactivated, and Δ*F*_*j*_ as the activation free energy.

By definition,

6 Δ*F*_*j*_ = Δ*U*_*j*_ − *T*Δ*S*_*j*_,

where 
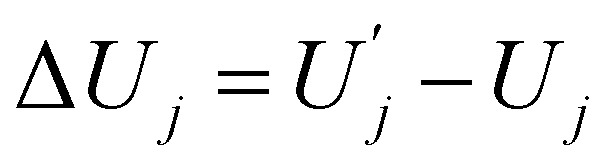
 and 
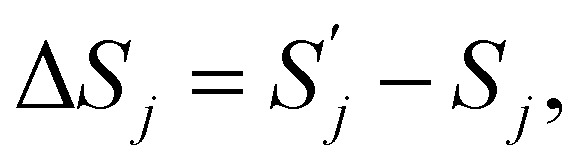
 are, respectively, activation energy and activation entropy.

To simplify we will assume the following:

7 *F*_*j*_ = *F*(*j* = 1,2,3,…),

8
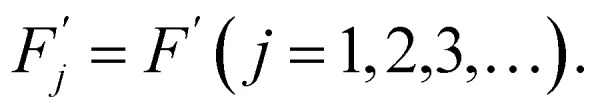


Thermally activated processes are assumed to take place in a system in the thermal equilibrium. In this case, the fluctuation behavior is governed by Boltzmann's principle^12^ and expressed as9
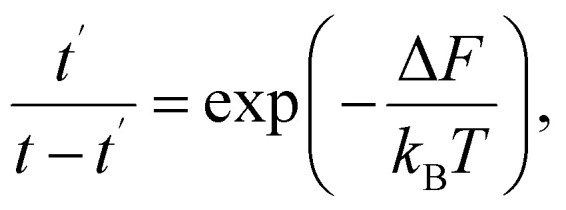
where *t* is the time of the observation and *t*′ is the total time during which the system exists in the state with free energy *F*′.

We will denote the mean lifetimes in states with free energies *F* and *F*′, respectively, as *τ* and *τ*′.

In sequence (1) the system undergoes a change from the state with free energy *F* to the state with free energy *F*′ and then back to the state with free energy *F* again and again. In other words, over a long enough interval, the number of times each of the states is detected is equal. Denoting this number as *q*, we have *t*′ = *qτ*′ and *t* − *t*′ = *qτ*. Consequently, we can rewrite (9) as10
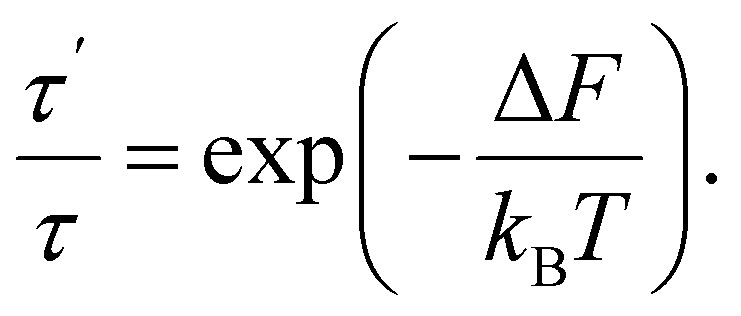


Substitution of the expression for the difference in free energies (6) yields11
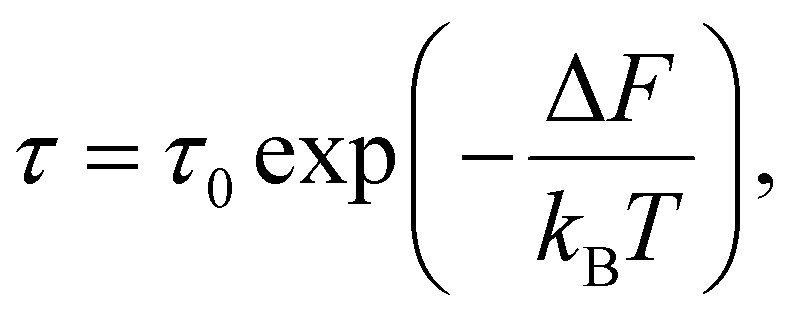
where12
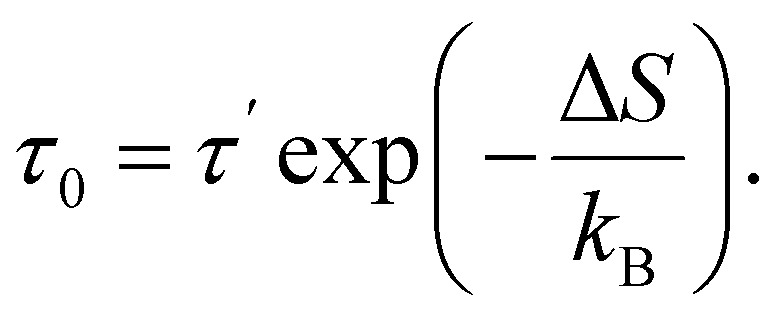


Expression (11) is widely referred to as Arrhenius equation.

Many researchers using Arrhenius equation to describe thermally activated processes were baffled by the anomalously small value of the pre-exponential time *τ*_0_. For instance, Meißner *et al.*^13^ determined the pre-exponential time for cellopentaose as *τ*_0_ = 10^−17^ s, that for chitosan as 10^−16^ s. The authors of ref. 9 state that the per-exponential time for salol in porous glasses is 1.8 × 10^−15^ s. Many works on the thermoactivated processes, do not state the values of the preexponential time at all, as (presumably small values) would be extremely hard to explain within the existing theoretical framework; see, for instance, ref. 14–16. Some authors attempt to explain such small values by suggesting the activation energy has both enthalpy and entropy contributions.^17^ Other authors, who obtained the values of *τ*_0_ for polyethylene glycols that turned out unusually high^18^ explained such result by the presence of the entropy input. The same explanation for the unexpectedly high values of *τ*_0_ was offered in ref. 19 (water–cellulose–NaCl systems), in ref. 20, (water solutions of the hydroxypropylcellulose), and in ref. 21 (systems with cellulose derivatives). Still, no model that would allow for the understanding why this entropy input is present, let alone quantitatively estimate it, exist. The present work outlines the development of a model for thermally activated diffusion processes that could directly tackle the difficulties described. In order to make this ambitious task manageable, we chose the thermally activated diffusion processes as the focus of our research.

### 1.2. The two traditional approaches to the microscopic mechanism of thermally activated processes

Microscopic theory of thermally activated processes^2,22^ in solids is rooted in the zeroth approximation of the adiabatic perturbation theory,^23^ according to which a solid is a collection of non-structured particles, the point force centers. The role of these particles could be played by atoms, groups of atoms, or molecules. In such a model the thermal motion is usually assumed to consist of only vibrations of particles about their equilibrium positions. However, the model also allows for the translational motion of a particle: if it gains sufficient energy (due to an interaction with neighboring particles), a particle can ‘jump’ into a new equilibrium position, moving through the distance *a*, the mean distance between the system's particles. Such ‘jump’ is usually referred to as an elementary translation and constitutes an elementary thermal fluctuation event.

The space near the particle that is about to jump must be significantly disordered. This space has two regions: a void of dimension *a* near the particle (a hole or a vacancy); this is the void into which the particle will shift during the elementary translation. The rest of this space is filled with the neighboring particles, the distance between which, *a*′, can be estimated as 0.1*a* < |*a*′ − *a*| < *a*.

Henceforth we denote the number of particles in the system as *N*, and the period and frequency of the *j*-th normal vibration of a particle as *θ*_*j*_ and *ν*_*j*_, respectively.

By definition,

13 *ν*_*j*_ ≪ *ν*_3*N*_,

where *ν*_3*N*_ is the upper limit of the frequency spectrum, whose value, according to ref. 23, is

14 *ν*_3*N*_ ≈ 10^13^ s^−1^.

We can then rewrite expressions (13) and (14) as:

15 *θ*_*j*_ ≥ *θ*_3*N*_,

16 *θ*_3*N*_ ≈ 10^−13^ s,

where *θ*_3*N*_ = *ν*_3*N*_^−1^ is the smallest possible period of normal vibrations.

**Fig. 1:**
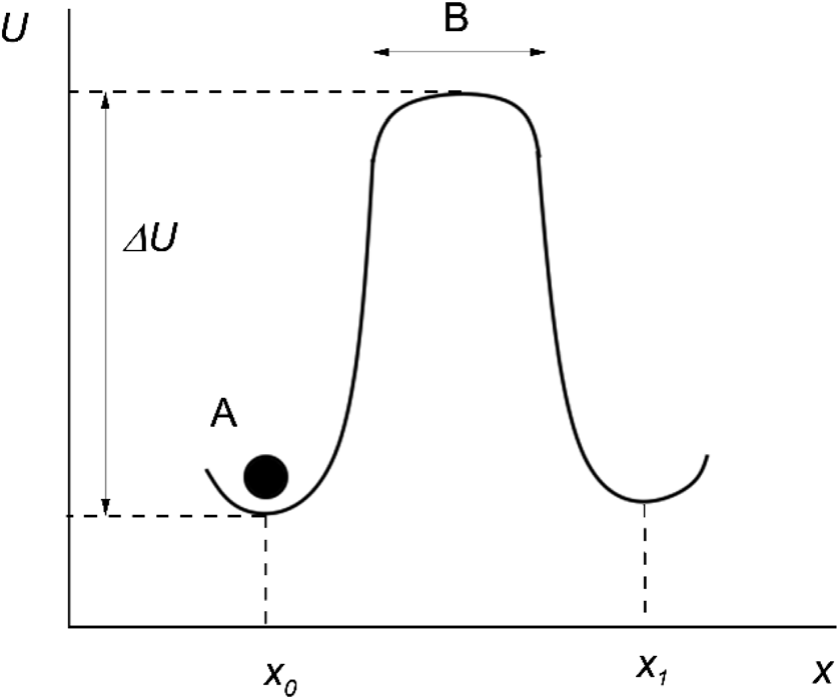
Particle's motion in the mean force field.

Originally, the microscopic theory of thermally activated processes was developed in the mean field approximation.^2^ According to this approximation, the particles around the “jumping particle–hole” pair are stationary, and the motion is assumed to take place in a constant mean force field generated by the surrounding of the pair. In this case, the potential energy of a particle *U* at different distances along the direction of motion of the particle *x* can be represented as shown in Fig. 1 (the moving particle is represented as a black circle and labeled A).

After several vibrations around an equilibrium position *x*_0_, the particle A jumps up the energy barrier Δ*U*, and continues to move rectilinearly along the *x*-axis at a speed equal to the mean speed of thermal motion.

The time the particle spends at the top of the barrier (or, in other words, the lifetime of the activated state for this model) is determined as:^2^17
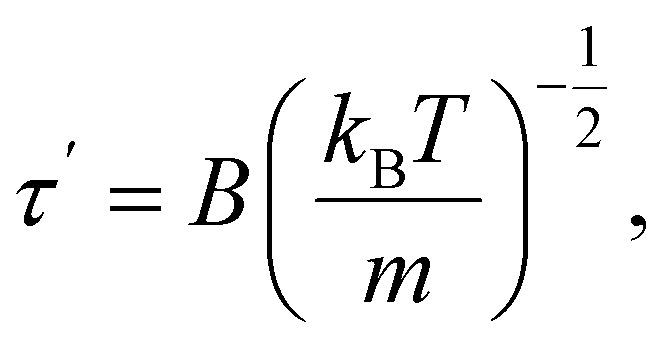
where *B* is the width of the energy barrier and *m* is the mass of the particle.

The time *τ*′ is usually estimated to be of the order

18 *τ*′ ≈ 10^−13^ s.

Thus, if we compare (16) and (18), the following is true:

19 *τ*′ ≈ *θ*_3*N*_,

In other words, the lifetime of the activated state should be approximately equal to the smallest period of normal vibrations.

Unfortunately, if we assume that the neighboring particles are stationary, it would be impossible to calculate free energy which characterizes an elementary thermal fluctuation event. This, however, becomes possible within the model described in ref. 22. In this model, the motion of the neighboring particles is taken into account.

Let's denote a vector whose components are *x*_*j*_ coordinates of particles as ***X*** ≡ {*x*_*j*_}. A point in this 3*N* dimensional space corresponds to a given configuration of particles of the system. We'll also denote the potential energy of interaction between the particles of this configuration as *W*(***X***).

Vineyard^13^ describes the transition from the stable inactivated state ***X***^(A)^ into a stable inactivated state ***X***^(B)^ through an activated state corresponding to a saddle point ***X***^(P)^. Near these points, the potential energy of the system of particles is given by20
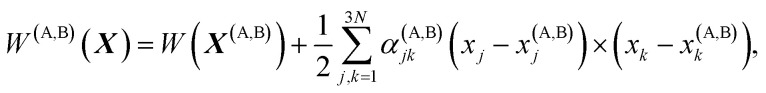
and21
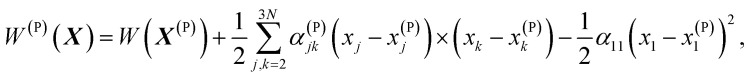
where22
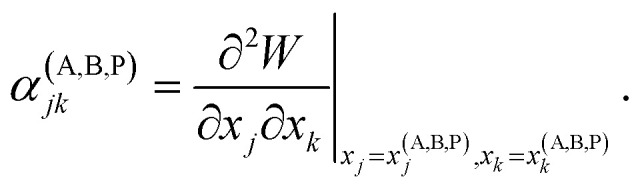


For each of these states Vineyard used traditional methods of statistical thermodynamics^23^ to calculate free energy, with the following condition applied when taking the statistic integral for the activated state:

23 *x*_1_ = *x*^(P)^_1_ = const.

According to the expressions (20)–(23), both activated and inactivated states in this model are viewed as the states of phonon equilibrium, or the states that correspond to the equilibrium between the vibrational degrees of freedom.

As suggested by in ref. 4–11, the approaches first formulated in ref. 2 and 22. still act as the basis for the current understanding of the microscopic theory of thermally activated processes.

## 2. Objective

The belief that an activated state is established *via* phonon mechanism is shared by many researchers. And yet, this mechanism description can lead to a very grave contradiction both with Frenkel's model^1^ and with the basic principles of thermodynamics.

Earlier we showed that eqn (23) states that a particle at the top of the energy barrier cannot begin its motion along the *x*_1_ axis until a phonon equilibrium, corresponding to the activated state is established. If *τ*_a_ is the time it takes to establish this equilibrium, then

24 *τ*_a_ ≫ *θ*_3*N*_.

The time the particle spends at the top of the energy barrier is essentially the lifetime of the activated state. Thus, if the phonon mechanism of activated state formation is at work, the following must be true:

25 *τ*′ ≈ *τ*_a_.

And, with (24) in mind

26 *τ*′ ≫ *θ*_3*N*_.

In other words, according to (23), a particle at the top of the energy barrier should remain stationary for a significantly longer time, governed by condition (26).

Such behavior is in direct contradiction with Frenkel's model,^2^ which suggests not only that the particle must spend a much shorter time at the top of the barrier (19), but it should also undergo translational motion.

Even if we do not invoke the reasoning outlined in ref. 2, expression (23) looks suspicious, as all particles of the system should be in a constant thermal motion, not temporarily ‘nailed down’ to certain locations.

Thus, if the phonon mechanism for the activated state formation is rooted in the assumption (23) and this assumption leads to several contradictions, one should conclude that the phonon mechanism is invalid. But what can serve as an alternative mechanism? A solid can be viewed as the collection of two subsystems: a phonon subsystem and an electron subsystem. Thus, if an activated state cannot be the state of the phonon subsystem, it must be the state of the electron subsystem. In the present paper, we set out to describe a possible mechanism of such state formation.

## 3. Theoretical model

### 3.1. Lifetime of the activated state

Particles' coordinates change over time, so the position vector ***X*** is the function of time:

27 ***X*** = ***X***(*t*).

and so is the potential energy:

28 *W* = *W*(***X***(*t*)).

By definition,^23^ potential energy *W* is the energy of the electronic subsystem in the stationary state with the lowest energy, the ground state. This state can be established over the mean time *τ*_e_. According to (28), this state should exist continuously at any instant *t*. The latter statement can only be true if

29 Δ*t* ≈ *τ*_e_.

where Δ*t* is the time scale used in (27).

Expression (28) implies that *t* is a continuous variable. Thus Δ*t* should be thought of as an infinitesimally small value:

30 Δ*t* → 0.

The behavior of the atom jumping from one equilibrium state into another is described in ref. 2 using a Poisson distribution. According to this distribution, the probability of no jump occurring over time *t* is given by31
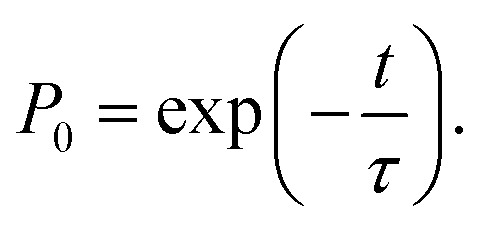


An important assumption made during the derivation of (31), is the equality^24^32
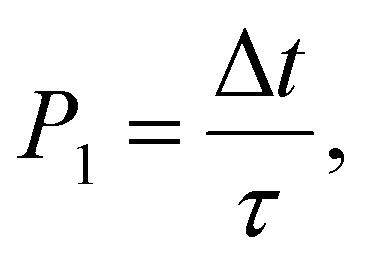
where *P*_1_ is the probability of the jump over time Δ*t*. Comparing (32) to (10), we can see that the latter defines the same probability. Thus,

33 *τ*′ ≈ *τ*_e_,

In other words, the lifetime of the activated state is approximately equal to the mean time during which the ground state in the electron subsystem is established.

When the smallest period of vibration *θ*_3*N*_ is introduced, it is simultaneously assumed that function (27) has different values at times *t*, smaller than this period:

34 *t* < *θ*_3*N*_,

From here it follows that

35 Δ*t* ≪ *θ*_3*N*_.

Comparing (29) and (35), we conclude that the mean time *τ*_e_ is smaller than the smallest period of vibrations:

36 *τ*_e_ ≪ *θ*_3*N*_.

Let *ν*_e_ denote the characteristic frequency of electrons, whose relation to *τ*_e_ is as follows:

37 *ν*_e_^−1^ ≪ *τ*_e_.

Using the value of *ν*_e_ estimated in ref. 23 as *ν*_e_ ≈ 10^15^ s^−1^ and considering expressions (16), (33), (36), (37), we deduce that the lifetime of the activated state must be between

38 10^−15^ s ≪ *τ*′ ≪ 10^−13^ s.

Consequently,

39 *τ*′ ≈ 10^−14^ s,

a value we'll use for the lifetime of the activated state.

### 3.2. Activation free energy

It is widely accepted that free energy characterizes the equilibrium state of a physical system. Thus, when an activated state is assigned certain values of free energy, we can assume that this state is an equilibrium macroscopic state.

As this state is established over a finite time *τ*_e_, the space region in which it is established is also finite. In other words, an activated state is a locally equilibrium state. We shall refer to this space region as an activation zone.

As stated earlier, when the zeroth approximation of adiabatic perturbation theory is applied, a group of atoms can be viewed as a structureless particle. The region of space occupied by these atoms is assumed negligibly small. This point is referred to as a force center.

Although we will not apply this approximation, we will still refer to such a group of atoms as a “particle”. Physics of polymers^25^ often treats a subunit of a polymer as a force center. We will borrow such an approach.

Now the particle in question has a finite size *a* and acts as a collection of nuclei and electrons.

The number of particles in the zone is denoted *Q*. The particles are assumed to be identical.

If the size of the activation zone is *L*, then the expression for *Q* is40
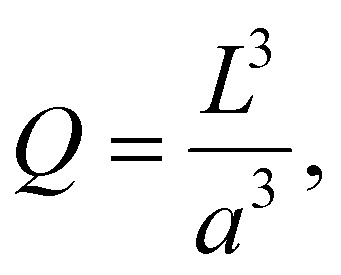


Electrons of all *Q* particles form an electron sub-system of the activation zone, the sub-system in which the activation state takes place.

The general expression for the free energy can be written as41
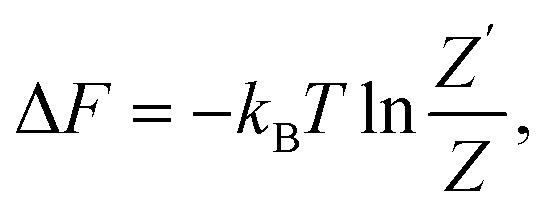
where *Z*′ and *Z* are the statistical sums of the electron subsystem in activated and inactivated states. These sums are determined by expressions:42
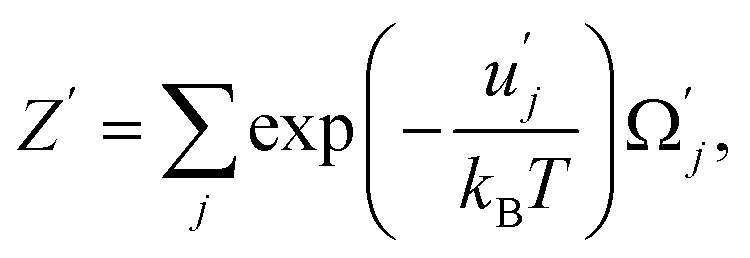
43
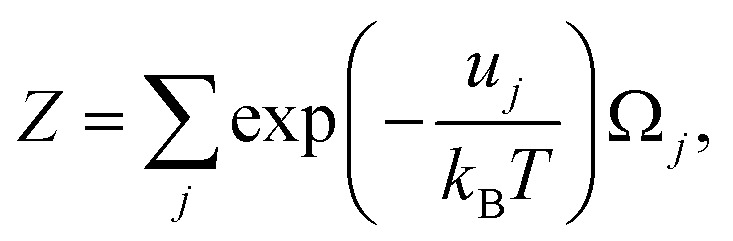
where 
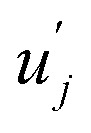
 and *u*_*j*_ are the energies of microscopic states comprising the macroscopic ones for, respectively, activated and inactivated states of the electronic subsystem, whereas 
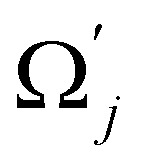
 and Ω_*j*_ – are the numbers of the microscopic states with energies 
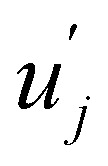
 and *u*_*j*_.

Let's now denote the position vector of a particle as ***R*** and the wave function corresponding to the energy level *E*_*α*_ of an isolated particle as *φ*_*α*_(***r*** − ***R***).

Due to the disordering of the activation zone, wave functions can be assumed to not overlap:44
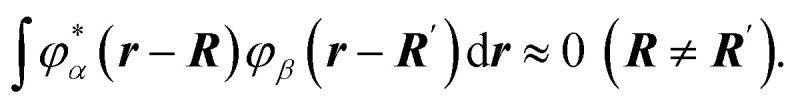


We will also assume that this condition stays true for all particles of the activation zone.

The particles of the activation zone absorb heat from the thermostat and vibrate. Due to the electron–phonon interaction, some electrons in the activation zone can be excited. Without going into specific details about the energy spectrum of the particle, we will refer to the ground state of the electrons as *ε*′ and the excited state as *ε*. We will assume the particles of the activated zone are not excited when their electrons subsystem is in an inactive state.

Consequently, eqn (43) can be rewritten as:45
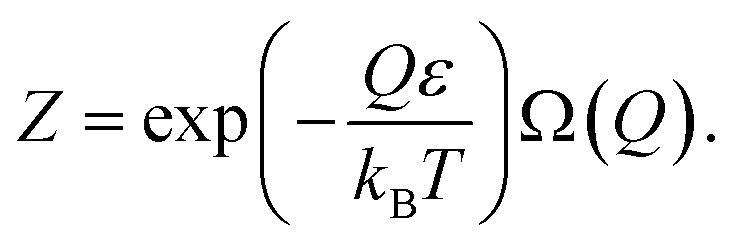


Excited particles transfer their energy to unexcited particles due to resonant interaction.^23^ This nonequilibrium process results in the formation of the equilibrium (and activated) state in the electron subsystem:46
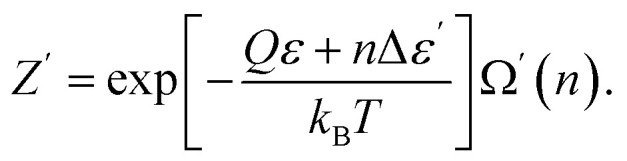
where *n* is the number of excited particles in the activated zone, and Δ*ε* = *ε*′ − *ε* – is the particles' excitation energy.

Substitution of (45) and (46) into (41) yields

47 Δ*F* = *n*Δ*ε* − *k*_B_*T* ln Ω′(*n*).

An activated state, like any other equilibrium state corresponds to the minimum of free energy. In our model, in which each of the excited atoms' location in the activation zone must have the same energy, the minimum of the free energy is attained when the entropy is the greatest. The entropy of the system attains its maximum value when the location of the excited particles is equally probable, making Ω′(*n*) the number of ways the excited particles are arranged in the activation zone:48
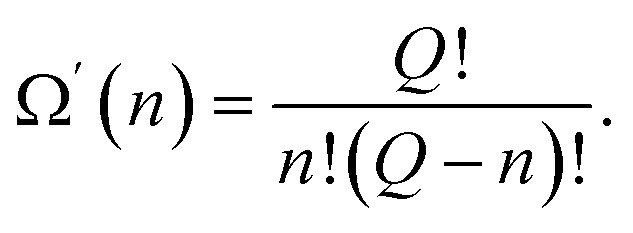


Consequently, according to the mechanism proposed, if we compare expressions (6) with (47) and (48), we can write the expressions for the energy and the entropy of activation as

49 Δ*U* = *n*Δ*ε*,

50
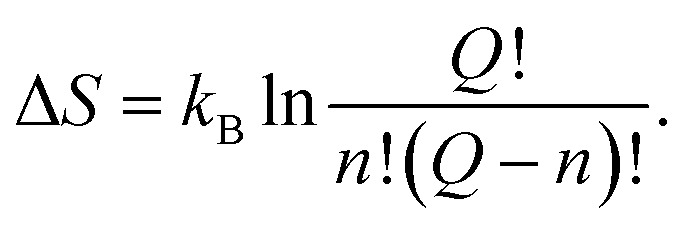


Thus, the present theoretical model implies the following mechanism of activated state formation.

The activated state is formed in a finite-sized region – the activation zone. The energy of this region is the sum of two terms: the vibrational energy of the particles that make up the region and the energy of the electrons that make up the particles. The first is, essentially, a set of vibrational elementary excitations – the phonons. It is thus referred to as the phonon subsystem. The other system is then the electronic subsystem.

Due to thermal fluctuations, the vibrational energy of the particles of the activation zone at a certain instant becomes much greater than the average vibrational energy. This increased vibrational energy is transferred to the electrons of particles of the activation zone by means of the electron–phonon interaction: in other words, the energy is transferred from the phonon to the electronic subsystem, and the activation zone gains excited particles.

Due to resonant interaction, these particles transfer their electronic excitation energy to other particles. This process continues until an equilibrium state is established in the electron subsystem, with excited particles evenly distributed across the activation zone. This state is the activated state.

## 4. Experiment

### 4.1. Outline of the experiment

Let's now experimentally validate the theoretical model outlined earlier. To do so we will examine the behavior of undecylenic acid nanocrystals in pores of the silica gel. The choice of this particular system is justified for the following reasons.

In the previous formulae we used the size of local equilibrium (activation zone) *L*. Strictly speaking, its value is determined by the process of equilibrium formation. To our best knowledge such a process for solids has not been sufficiently elucidated in research. The determination of *L* for this system is significantly simplified. Firstly, it is evidently true that

51 *L* ≤ *D*

where *D* is the size of the nanocrystal.

Secondly, the theoretical model does not foresee the interaction between the processes taking place in different regions of the local equilibrium. Such property of model is easily realized for the nanocrystals in silica gel pores, as the silica gel matrix is much more rigid than nanocrystals. Thus, during the relaxation process, a given nanocrystal does not deform the matrix, and thus the rest of the nanocrystals in other pores do not “feel” what happens in other parts of the system. The actual chemical composition of nanocrystals in the pores – be it undecylenic acid or any other substance – is of no importance here; what matters is that they should not be able to deform the matrix. By the same logic, it doesn't matter whether the pores in the matrix are ordered or unordered. Thirdly, the absence of the interaction between the relaxation processes, taking part in different nanocrystals, allows us to introduce model,^26^ which is based on the elasticity theory, and which makes it possible to calculate *L* from experimental data obtained when observing relaxation processes in nanocrystals.

As stated earlier, for the relaxation process to take place, the activation zone must be disordered, *i.e.* it must have structure defects. The locally equilibrium state in the electron subsystem within the activation zone is possible only due to the presence of such defects. Essentially, the whole activation zone can be treated as a defect, and the ratio *L*/*D* can serve as the measure of the defect presence in the nanocrystal.

The authors of ref. 26 state that *L* is independent of the size of the nanocrystal; in other words, as *D* decreases, the degree of defect presence increases. Such result agrees with a well-established idea that smaller nanocrystals are more defect-ridden.

Among the methods applied to study thermally activated processes, mechanical and dielectric relaxation methods occupy a prominent place.^27–32^ Out of these two, the rigidity of the silica gel matrix in which nanocrystals are located precludes the use of the former.

The dielectric spectroscopy method examines the behavior of the complex generalized permittivity ε*(*ω*_e_,*T*) at different temperatures *T* and different frequencies *ω*_e_ of the external field.

To estimate parameters of the thermally activated process we apply the following algorithm.

(1) A peak on the imaginary part *ε*′′(*ω*_e_,*T*) of ε*(*ω*_e_,*T*) indicates the presence of the thermally activated process. Denoting the temperature corresponding to this peak as *T*_e_ we can relate the frequency of the internal field to the temperature of the activated process.

52 *ω*_e_ = *f*(*T*_e_).

The values of the parameters *τ*,*τ*_0_ and Δ*U* are then determined from the well-known expression

53 *ω*_e_*τ*(*T*_e_) = 1,

and from54
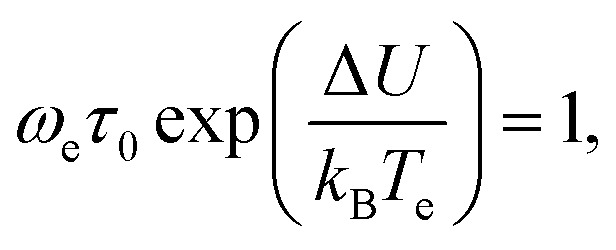


which is obtained by substitution of (53) into (11).

(2) Using the calculated value of *τ*_0_, as well as an expression (12) and an estimate (39), activation entropy Δ*S* is then calculated.

(3) Using expression (40) we can then determine the number of particles in the activation zone *Q*.

(4) A subsequent substitution of Δ*S* and *Q* into (50) yields the number of excited particles *n*.

(5) Finally, we can deduce the value of the particle's excitation energy Δ*ε* by substituting values of *n* and Δ*U* into expression (49).

### 4.2. Materials and methods

Undecylenic acid was purchased from Sigma-Aldrich (≥96%, CH_2_=CH(CH_2_)_8_COOH). Three different silica gels with varying pore sizes (KSK 2.5 and KSS 4 and Silica Gel 60 (fraction 0.063–0.2 mm) from UCT, USA) were selected as nano-porous matrices. Large particles of KSK 2.5 and KSS 4 were ground, and a 0.1 to 0.35 mm size fraction was sieved. The resulting particles were boiled in 70% HNO_3_ for 4 hours to remove impurities, then thoroughly washed with deionized water, and finally dried in air at 110 °C. The sizes of pores in silica gel were estimated as^33,34^*D* = 16.9 nm for KSK 2.5, *D* = 10.6 nm for silica gel 60 and *D* = 6.8 nm for KSS 4.

To prepare the nanocomposites of silica gels with undecylenic acid (SiO_2_/C_11_H_20_O_2_), the SiO_2_ samples were mixed with a 20% solution of undecylenic acid in hexane; the quantity of undecylenic acid taken filled between 75% and 80% of the total pore volume. The resulting suspensions were sonicated for 5 minutes to remove air bubbles from the nanopores. Afterward, the suspensions were first left to dry under ambient conditions with periodic agitation and then dried at 90 °C. The samples obtained were free-flowing white powders, whose particles did not stick to each other.

The dielectric properties in the frequency range from 1 kHz to 50 kHz and the temperature range from −196 °C to 100 °C were studied on an automated installation based on a P5083 AC bridge and a four-electrode thermostatted cell, with an option of a sample thickness control.^35^

The pore size and surface areas of silica gels were measured by N_2_ adsorption at 77 K, using Sorptometer KELVIN 1042. The isotherms were treated by standard BET and BJH algorithms, using the software provided by the Sorptometer manufacturer.

## 5. Results and discussion

### 5.1. Dielectric spectroscopy

**Fig. 2:**
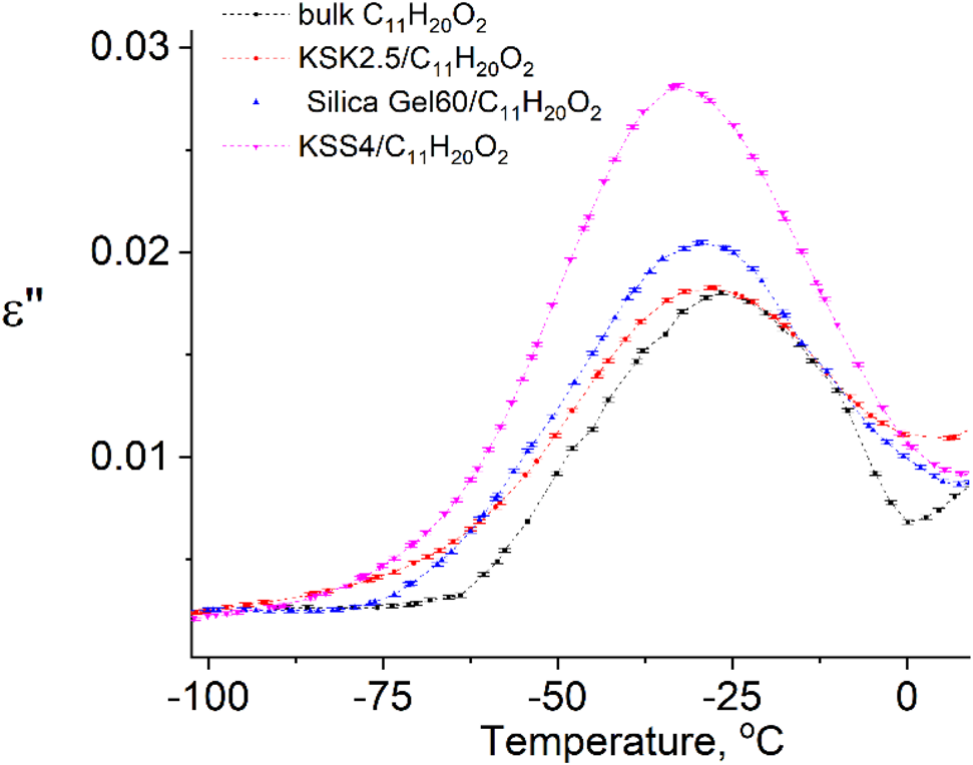
Temperature dependencies of the imaginary part of dielectric permittivity of samples at *f* = 50 kHz.

**Fig. 3:**
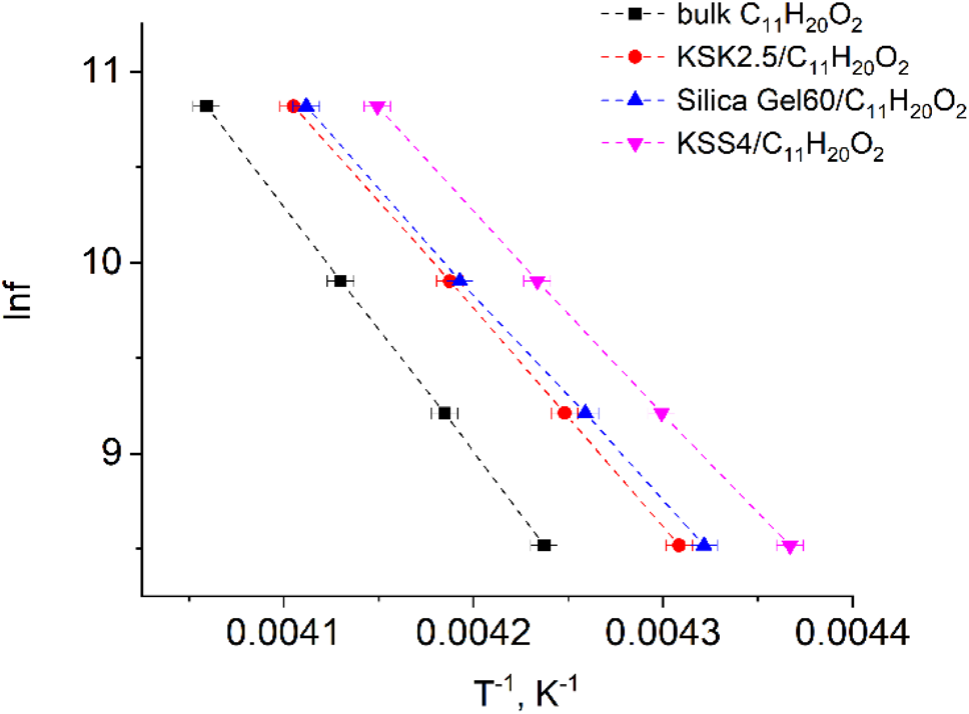
Natural logarithm of electric field frequency *vs.* inverse temperature *T*_e_ corresponding to the peak in the imaginary part of dielectric permittivity of samples.

In our previous work^36–39^ we have presented the graphs of the real *ε*′ and imaginary *ε*′′ parts of the complex dielectric permittivity ε*(*T*,*f*) *vs.* temperature for the bulk undecylenic acid and nanocrystals of the undecylenic acid in the silica gel pores of different dimensions. Some of the results obtained earlier are shown in Fig. 2 for reference: this figure describes the behavior of the imaginary part of dielectric permittivity at different temperatures for undecylenic acid in bulk and in the nanocrystalline form (electric field frequency 50 kHz). All the samples undergo a dielectric relaxation. The dielectric relaxation shifts to the lower temperatures as the nanocrystal size decreases. The data in Fig. 2 can also be processed to determine the relationship between the frequency of the external field *f* (were *f* = *ω*_e_/2π) and the temperatures of maxima *T*_e_. It is represented on a ln *f vs.* 1/*T*_e_ graph in Fig. 3.

The points in Fig. 3 are well-fitted with straight lines, indicating that the relationship between ln *f* and *T*_e_^−1^ is described by expressions (54) and (11). In other words, the above-described maxima are observed due to the presence of the thermally activated process.

All parameters describing the activation process are calculated from data in Fig. 3 using expressions (54), (11), (12), (39), (40), (49), and (50). The values of these parameters are summarized in Table 1. The value of the activation zone *L* used was 4.6 nm as calculated in ref. 26. The particles taking part in the activation process were the links of the undecylenic acid chain corresponding to *a* = 0.5 nm. The values of *E*(*n*), listed in the table, describe the mathematical expectation for the number of particles *n* excited in the activation zone. They were calculated by comparing the calculated and the experimental values of the activation entropy.

**Table 1:** Parameters of the thermally activated process in the nanocrystals of undecylenic acid

	*τ*_0_, s	Δ*S*/*k*	exp(Δ*S*/*k*)	*Q*	*n*	Δ*U*, kJ mol^−1^	Δ*ε*, kJ mol^−1^
Bulk C_11_H_20_O_2_	7.0 × 10^−29^	32.6 ± 0.5	1.4 × 10^14^	780	5.9	107 ± 1	18 ± 1
KSK2.5/C_11_H_20_O_2_	1.9 × 10^−26^	27.0 ± 0.5	5.3 × 10^11^	780	4.7	94 ± 1	19 ± 1
Silica gel 60/C_11_H_20_O_2_	9.3 × 10^−26^	25.4 ± 0.5	1.1 × 10^11^	780	4.4	91 ± 1	23 ± 1
KSS4/C_11_H_20_O_2_	2.5 × 10^−25^	24.4 ± 0.5	4.0 × 10^10^	780	4.3	88 ± 1	22 ± 1

**Fig. 4:**
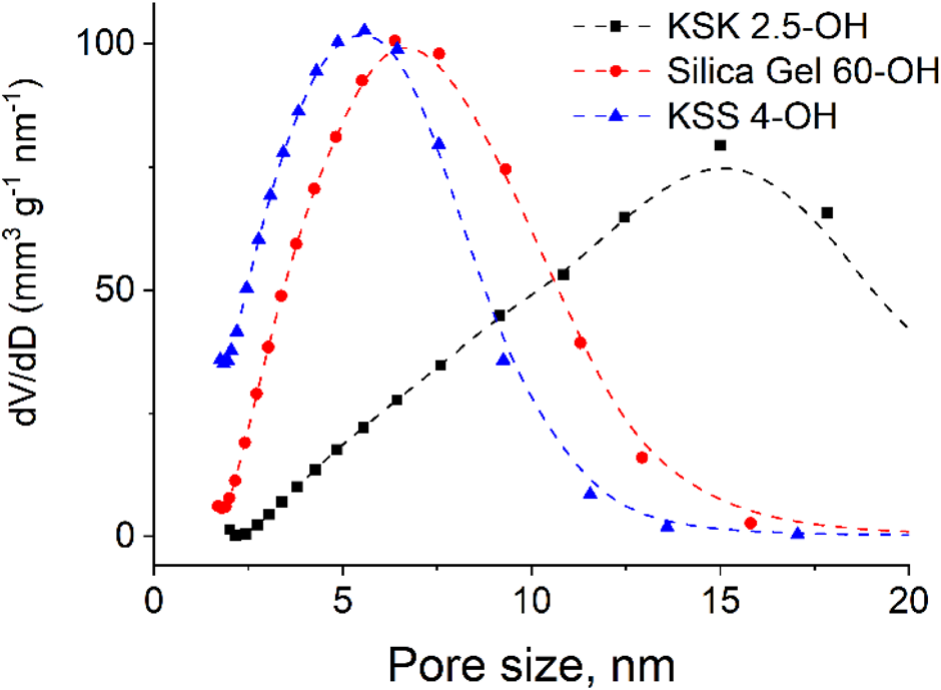
Pore size distribution in silica gels: a – KSK 2.5, silica gel 60 and KSS4 nanocomposites (obtained using N_2_ adsorption at 77 K on Sorptometer KELVIN 1042).

**Fig. 5:**
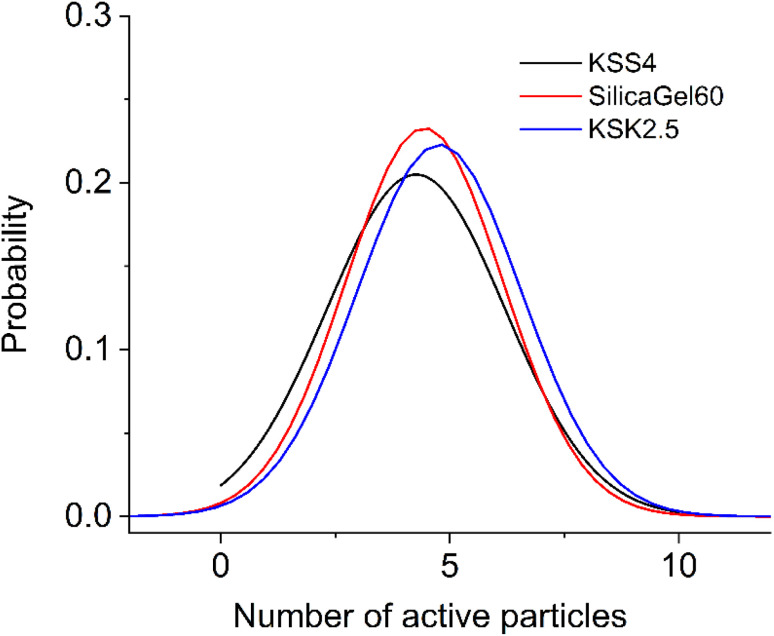
Distribution of excited particles in the activation zone (10^5^ particles): KSS 4 – *σ* = 1.947, SilicaGel60 – *σ* = 1.714, KSK 2.5 – *σ* = 1.790.

Since the sample contains pores of various sizes (Fig. 4), there should be a corresponding distribution of the excited particles present in the activation zone (as we have hypothesized that this number of particles must be affected by the size of the pore). We assumed that the number of particles in the activation zone in each sample obeys normal distribution (Fig. 5). We also applied the following condition: the coefficient of variation is identical in the excited particle number distribution and in the pore size distribution. When calculating the mathematical expectation, we used the following condition: the calculated value of the activation entropy (obtained using expression (50)) must be equal to the experimental value of Δ*S*. Following these computations, we have determined the mathematical expectations *n* of the number of excited particles in the activation zone, which are listed in Table 1.

**Fig. 6:**
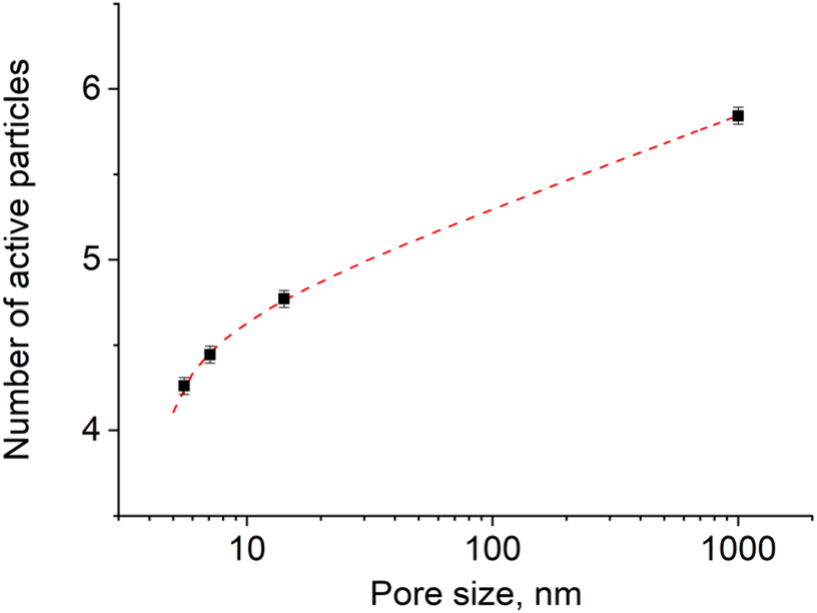
The variation of the number of excited particles in the activation zone with the size of pores (sample size is 10^5^ particles) in logarithmic scale.

We generalized these results to obtain an algebraic expression relating the value of *n* and the pore size *r*. By fitting the experimental results with a logarithmic function (Fig. 6, adjusted coefficient of determination *R*^2^ = 0.99945) we obtained the following expression:

55 *n* = 4225 + 0.235 ln(*r* − 4.403).

### 5.2. Discussion

To verify whether the experimental data agrees with the electron mechanism outlined in the previous sections, we will interpret and explain the results listed in Table 1.

(1) The value of *τ*_0_ is found to be between 10^−25^ and 10^−28^ s.

This is a well-known problem in the physical chemistry of thermally activated state, which can be referred to as the “Arrhenius term *τ*_0_” in eqn (11). It has a long history, being referenced in many papers.^13–21^ In the nutshell the problem is as follows: since most researchers describe the activation state formation in the framework of the phonon mechanism, they treat the system as a collection of structure-less particles, atoms or small groups of atoms. Then, if *τ*′ ≈ 10^−13^ s, the calculations, (*e.g.* ref. 2) of the activation entropy give the values for Δ*S* = *k*_B_, which then, using (12) yields *τ*_0_ < 10^−14^ s.

However, the values of *τ*_0_ determined experimentally are very far from that theoretical prediction of 10^−14^ s. For instance,^9^ reports the value of *τ*_0_ to be 10^−15^ s, whereas the authors of ref. 13, mentioned in the introduction, report the values between 10^−16^ and 10^−17^ s.

Usually, in order to resolve this contradiction, researchers^40^ assume the non-linear behaviour of *τ*_0_ at different temperatures. However, they fail to address what molecular mechanism causes such behaviour.

The electronic mechanism we presently propose, however, can resolve this contradiction. As Table 1 suggests, the values of activation entropy, calculated using the formulae derived for the electronic model, are 20–30 *k*_B_. If substituted into (12), they then give the value of *τ*_0_ listed in Table 1.

(2) Activation energy decreases with decreasing size of the nanocrystal.

As outlined earlier, the activation zone is quite disordered. The presence of defects in the structure is known^41^ to lead to the localization of oscillations. Thus, the vibrations of the phonon subsystem can be treated as localized. These oscillations absorb energy, and *via* interaction with normal modes spread through the nanocrystal.

If the nanocrystal is smaller, it has smaller number of such oscillations. Hence, the number of elementary interactions between these oscillations and oscillations localized in the activation zone decreases. This leads to the decrease in the energy entering the phonon subsystem of the activation zone. Moreover, it decreases the energy transferring from the phonon to electronic subsystem, the latter, according to the model proposed here, is the activation energy.

(3) Activation entropy decreases with decreasing sizes of the nanocrystal

By definition, the electronic subsystem gains energy from the phonon subsystem *via* quanta of energy *hν*, where *ν* is the phonon frequency.

As the activation zone is small and the oscillations are localized, their frequencies must be close to its limiting value of 1/10^−13^ s, leading to *hν* = *h*10^13^ J. As Table 1 suggests, each excited particle carries the energy of *h*10^13^ J (within experimental error) and the number of excited particles *n* is smaller than the total number of particles *Q* in the activation zone. The latter fact is easy to explain: the energy of the particle's excitation can not be smaller than *h*10^13^ J and thus the energy Δ*U* entering the electronic subsystem is not high enough to excite all particles of the activation zone.

As the energies of particle excitation are identical, the number of the excited particles *n* must be proportional to the total excitation energy, Δ*U*. Consequently, if Δ*U* decreases, so must *n*, and the activation entropy Δ*S* must decrease as well.

## 6. Conclusion

By definition, a thermally activated process is a succession of non-activated and activated states. A consensus nowadays is that the activated state is the locally-equilibrium state of the phonon subsystem. This statement, however, is self-contradictory: on the one hand, the lifetime of the activated state equals the smallest period of vibrations. On the other hand, as this equilibrium is established due to the interaction with normal vibrations of the molecules – the collision of phonons – the time interval between these collisions must be much greater than the period of vibrations. In other words, the lifetime of the activated state must be much greater than the period of vibrations. With this contradiction in mind, the activated state in a phonon subsystem simply cannot appear.

An activated sate is the locally equilibrium state of the electron subsystem. This subsystem consists of electrons of the particles that are part of the activation zone. These particles are groups of atoms acting as the force center in the adiabatic approximation.

The mechanism of the activated state realization can be summarized as follows: vibrational energy of the particles is transferred to the electrons of these particles *via* the electron–phonon interaction. The value of this energy is not sufficient to excite electrons of all particles of the activation zone, so only some particles are excited. They transfer the excitation energy to other particles of the activation zone *via* a resonant mechanism. This non-equilibrium process leads to the establishment of the local equilibrium state in which different positions of the excited particles in the activation zone become equally likely. Such a state is the activated state. The free energy of the thermally activated state is the free energy of this locally equilibrium state of the electron subsystem of the activation zone.

## Data availability

The data supporting this article have been included as part of the ESI.†

## Author contributions

M. M. Lazarenko – investigation, writing – original draft, supervision, project administration; Yu. F. Zabashta – conceptualization, writing – original draft; A. N. Alekseev – methodology, S. A. Alekseev – investigation; K. S. Yablochkova – writing – review & editing; L. Yu. Vergun – visualization; D. A. Andrusenko – investigation, data curation; K. V. Cherevko – formal analysis; V. B. Shevchenko: investigation, validation; Roman V. Dinzhos – investigation; L. A. Bulavin – writing – review & editing.

## Conflicts of interest

There are no conflicts of interest to declare.
